# Commentary on ‘Quantifying conditions for climate control on coals and evaporites’ by Bao *et al.*

**DOI:** 10.1093/nsr/nwad111

**Published:** 2023-04-21

**Authors:** Judith Totman Parrish

**Affiliations:** Department of Earth and Spatial Sciences, University of Idaho, USA

Research on deep-time paleoclimate involves three complex lines of inquiry: reconstructing the geography of the Earth, climate modeling, and understanding just what paleoclimate proxies are recording. Once plate tectonics became widely accepted, most of the effort went into reconstructing the geography of the Earth. The studies that incorporated climate proxies into the research, for example, Briden and Irving [1] did so without critiquing very deeply what those proxies might mean, which was entirely appropriate given the state of the science at that time. Along with the assumption that we knew, at least in general, the significance of climate proxies were additional assumptions, which remained to be tested, about climate patterns and how they changed with the changing geography.

By the late 1970s–1980s, global geography was relatively well known, and formal climate models began to be employed. This was significant because, first, this allowed paleoclimatologists to investigate climate patterns independently of the distribution of the climate proxies, and, second, comparison of model predictions with climate proxies highlighted potential problems with the climate models, the reconstructed geography, and/or the proxies themselves. All three problems were revealed in various studies. For example, western equatorial Pangea was predicted to be very dry in the Triassic [2], but the geological record revealed that precipitation was extremely seasonal [3]. Discovery of that discrepancy opened an entirely new line of research on the effects of monsoonal climate on the geologic record. As reconstructions of the geography improved, so did the predictive strength of the models (e.g. [4]).

Perhaps most difficult has been understanding exactly what the climate proxies mean. The formation of coals, evaporites, paleosols, and the like is complex, and not just dependent on climate. For example, coal will not form in the absence of sufficient accommodation space for the developing peat bogs. Eolian sand dunes may form in interior dry deserts or on less-dry coasts, so without further context, the assumption that eolian sand dunes represent dry climates might be mistaken. Regardless, pattern matching between the distribution of climate proxies and predicted climate patterns helped push forward the understanding of the evolution of global climate.

The earliest quantitative climate proxies were stable isotopes, and they were developed in sufficient quantity to make comparison with the quantitative output of numerical climate models possible (e.g. [5] and subsequent work), particularly for study of the oceans (e.g. [6]). Quantification of the climate significance of continental climate proxies lagged somewhat, and depended on comparing modern analogs to coal, evaporites, vertisols, etc., to modern climate information (e.g. [7]). The early efforts of conceptual climate modeling and comparison with continental proxies were not without success. For example, the work anticipated Bao *et al.*’s finding that coals shifted from predominantly equatorial to high-latitude settings [2].

Bao *et al.*’s [8] work represents a significant step in the quantification of continental paleoclimate proxies. The improvement in paleogeographic reconstructions permits a much-finer division of the geologic record and a more-precise match of the proxies and climate models. The ecological explanation for the shift in coals expands the understanding of the evolution of this complex paleoclimate proxy. The overlap between evaporites and coals in the relationship with mean annual precipitation is intriguing, but deftly addressed by reexamining the data in light of precipitation/evaporation ratios. This work provides a firmer quantitative foundation for the evaluation of global climate change in deep time using these proxies.

The data also point to some interesting avenues for future research if the evaporites and coals that fall well outside the usual climate conditions are evaluated. What can we make of the outliers in their data? As with the too-dry western equatorial Pangea, the anomalies often contain the most interesting questions. And the surprisingly narrow and consistent climate conditions for these deposits indicate that the role of climate in enhancing coal formation, in particular, was stronger than previously appreciated.
